# Verbal learning in frontal patients: area 9 is critical for employing semantic strategies

**DOI:** 10.1007/s10072-024-07569-7

**Published:** 2024-05-09

**Authors:** Alessandro Cocuzza, Giulio Bertani, Giorgio Conte, Edoardo Nicolò Aiello, Barbara Zarino, Teresa Difonzo, Stefano Zago, Leonardo Tariciotti, Claudia Gendarini, Elena Baratelli, Federico Verde, Barbara Poletti, Nicola Ticozzi, Mauro Pluderi, Marco Locatelli, Giacomo Pietro Comi, Maria Cristina Saetti

**Affiliations:** 1 Department of Pathophysiology and Transplantation, University of Milan, 20122 Milan, Italy; 2 Foundation IRCCS Ca’ Granda Ospedale Maggiore Policlinico, Neurology Unit, 20122 Milan, Italy; 3 Foundation IRCCS Ca’ Granda Ospedale Maggiore Policlinico, Neurosurgery Unit, 20122 Milan, Italy; 4 Foundation IRCCS Ca’ Granda Ospedale Maggiore Policlinico, Neuroradiology Unit, 20122 Milan, Italy; 5 Department of Neurology and Laboratory of Neuroscience, IRCCS Istituto Auxologico Italiano, Milan, Italy; 6 Humanitas Psico Medical Care, MCH SRL, Via Manzoni 113, 20089 Rozzano, Milan, Italy; 7 Department of Neurology, G. Salvini Hospital, Garbagnate Milanese, Italy; 8 Department of Oncology and Hemato-Oncology, Università degli Studi di Milano, Milano, Italy

**Keywords:** Strategic memory, Semantic clustering, Verbal learning, Prefrontal cortex, Area 9

## Abstract

**Introduction:**

Learning is a long-term memory process heavily influenced by the control processes implemented by working memory, including recognition of semantic properties of items by which subjects generate a semantic structure of engrams.

**Aim:**

The aim of this study is to investigate the verbal learning strategies of patients affected by a tumor in the left frontal lobe to highlight the role of area 9.

**Method:**

Ten patients with frontal low-grade gliomas and ten healthy control subjects, matched for age, sex and education, were recruited and then evaluated with a two-part verbal learning test: multi-trial word list learning in free recall, and multi-trial word list learning preceded by an explicit semantic strategy cue. Frontal patients were divided into two groups: those either with frontal lesions involving or sparing area 9.

**Results:**

In comparison to healthy control subjects, frontal patients with lesions involving area 9 memorized fewer words and displayed difficulty in using semantic strategies. When the strategy was suggested by the examiner, their performance improved, but to a lesser extent than the healthy control. Conversely, frontal patients with lesions sparing area 9 showed similar results to healthy control subjects.

**Conclusion:**

The results suggested that, while the identification of the categorical criterion requires the integrity of the entire dorsolateral prefrontal area, only area 9, and not the surrounding areas, could be responsible for the effective use of semantic strategies in learning tasks.

## Introduction

Learning is a complex function that involves both short and long-term memory. It is achieved through typically mnesic processes (encoding, storage, recall) but also through regulatory processes including the implementation of learning strategies.

When a verbal learning test is administered to normal subjects, they tend to implement clustering strategies [1, 2] to improve items recall. If the learning material consists of a number of words pertaining to different semantic categories, the amount of recalled words correlates with subjects’ inclination to group those words by semantic category [3, 4].

Prefrontal lesions seem to impair the ability to figure out criteria for effectively organizing the items that have to be encoded, undermining the strategical retrieval of engrams from the long-term memory store [5–7]. Even though frontal patients struggle to spontaneously rely on semantic organization criteria, their performance in word list learning tasks [6–9] benefits from semantic cues when explicitly suggested [6, 8, 10]. This could mean that frontal patients are generally impaired in figuring out an appropriate learning strategy, and that the strategic cue may compensate for this deficit.

In less recent literature, distinctions between lesion sites in the frontal cortex were either not carried out [6, 9, 11–13] or, whenever applied, they were based on the damaged hemisphere (right side vs left side) [14–16]. The association between frontal injury sites and patterns of impairment began to be explored further from the 1990s. A fundamental contribution came from an early study by Stuss and colleagues [7], in which the authors subdivided frontal patients into subgroups based on lesion site. Stuss and colleagues demonstrated the association between damage in different frontal areas and patterns of impairment, suggesting the involvement of distinct neural mechanisms. Addressing the discrepancies among various studies and their corresponding interpretations of learning impairment in frontal lobe damage, Alexander and colleagues [17] emphasized that there is no single, homogeneous frontal lobe syndrome in relation to memory impairment. The authors demonstrated that different subgroups of frontal patients showed different performances in the California Verbal Learning Test. They also underlined that, out of all these subgroups, only patients with lesions in the left posterior dorsolateral frontal region or in the posterior medial one exhibited an impairment in learning processes. On the basis of the interpretation of Alexander and colleagues, it is important to highlight that previous discordances between studies on verbal learning in frontal patients may originate from the lesion site being a somewhat confounding factor: the most represented lesion site may determine the main features of memory impairment, leading the authors to assign a specific deficit (and function) to the whole dorso-lateral prefrontal cortex. On the other hand, effects of a lesion in a critical site might be mitigated by a certain number of frontal patients with lesions in non-critical sites.

## Dorsolateral prefrontal cortex

While item retention in short-term memory (STM) is related to the temporal [18] and parietal lobes [19], concurrent item manipulation (working memory, WM) correlates better with PFC activity.

According to Petrides [20–23], frontal lobes exchange information with posterior regions through bi-directional connections between the posterior cortical associative areas and the ventrolateral PFC (VLPFC), connected to the mid-dorsolateral PFC (DLPFC). From this perspective, VLPFC acts as the middle station that mediates the interconnection between posterior areas and DLPFC, thus playing an important role in second-level memory processes, such as the intentional retrieval of information from the long-term memory store. Instead, DLPFC is fundamental in higher memory functions such as active item manipulation and monitoring of already learnt information, as underlined by fMRI studies [24].

The use of reorganization strategies, carried out by the DLPFC, is based on the recognition of specific properties of items and enhances learning by strengthening the association between items in the long-term memory store [25]. Hence, DLPFC should be considered as a neural system not only able to encode new memories, but also able to contribute to the retrieval of old ones [26], improving engram consolidation and learning.

We refer to the WM component which selects and elaborates specific properties of items as “strategic memory”.

Alexander and colleagues [5] evaluated that critical lesions—in terms of a stronger association with impairments in learning and strategy implementation (i.e., a failure to use semantic organization)—were mainly located in the left superior frontal lobe (which they referred to as “area 9 s”). They proposed that left superior lesions may compromise the process of strategy implementation, supporting this hypothesis with the evidence [27] that temporary alterations in area 9’s activity, by means of repetitive transcranial magnetic stimulation, impaired the organization of verbal encoding.

Issues in defining a specific cognitive role of Brodmann’s area 9 (BA9), as a component of the PFC, mostly come from the adoption of different classification systems for cortical areas, and the variety of experimental protocols utilized over decades of research.

At first, Brodmann outlined his well-known classification of brain areas, in which he located area 9 in both the superior frontal gyrus and the middle frontal gyrus and, for the most part, subsequent studies on frontal functions were based on this formulation. However, addressing discrepancies between Brodmann’s classification of human brain areas and Walker’s corresponding classification for macaque monkeys [28], Petrides and Pandya [29] were able to develop a modern classification for BA9. The authors suggested a further breakdown of the original BA9 based on cytoarchitectonic properties in area 9/46 (in the middle frontal gyrus) and area 9 (in the superior frontal gyrus). Here, area 9 is restricted to the dorsomesial component of the anterior frontal cortex, laying on the superior frontal gyrus and extending medially to the paracingulate sulcus [29]. For the sake of clarity, the term “area 9 (A9)” will henceforth indicate the region described in Petrides and Pandya’s studies, therefore excluding area 9/46.

The aim of our study is to demonstrate that implementation of semantic clustering strategies in frontal patients is compromised if the lesion involves a specific part of the DLPFC, corresponding to A9. In this case, frontal patients do not take advantage of the categorical cue.

## Materials and methods

### Participant sample

Ten frontal lobe-damaged patients (FL) and 10 healthy control subjects (HC) were included in this study.

Subjects with frontal neoplastic lesions (low-grade gliomas) were recruited during a nine-month period from patients at the Neurosurgery Unit of the “Policlinico Maggiore” Hospital in Milan. Each patient was tested at least six months after surgery, concurrently with the control MRI scans.

The main inclusion criteria required a single primitive frontal lesion on the left prefrontal cortex. Patients had to be in treatment with anti-epileptic therapy and with no evidence of epileptic seizures in the past month. We considered the following exclusion criteria: brain lesions located in any site other than the left frontal lobe; neurodegenerative disease or any other neurological condition determining a history of cognitive decline; history of any other significant neurological or psychiatric disorder; non-neurological comorbidities that could potentially compromise the neuropsychological testing results. Patients had to obtain normal scores in MMSE [30], Token Test [31], Boston Naming Test [32]. Eleven FL were recruited. However, one female patient was excluded because of aphasia documented by significantly low scores in fluency testing and in the Token test. Therefore, the total number of FL patients eventually included in the study is ten, two males and eight females (average age = 48.7 ± 13.3 DS; average education 14.1 ± 3.8 DS).

Frontal patients were divided into two groups: 6 subjects with tumoral lesion sparing area 9 (Fn) and 4 subjects with lesion involving area 9 (F9).

For this purpose, three independent skilled operators were required to identify area 9 according to Petrides and Pandya criteria in the MRI sequences, evaluating whether it was spared by the lesion. The three operators’ judgements were unanimous, except for one FL patient whose circumstance was discussed until reaching an agreement. The neuropsychological testing was performed within 3 days before MRI.

Ten healthy control subjects (HC), matched to FL for age, gender, and years of education, were recruited for the experiment. Their medical history was negative for neuropsychiatric disorders and for the use of psychotropic drugs. Their performances in MMSE [30], Token Test [31] and Boston Naming Test [32] resulted within normal range.

### MRI acquisition

MRI examination was performed by a 3 Tesla Scanner (Philips Achieva Eindhoven, The Netherlands), equipped with a 32-channel sensitivity encoding coil. DTI sequence was acquired as an axial, single-shot, echo planar imaging sequence with the following parameters: repetition time = 7723 ms, echo time = 70 ms, flip angle = 90°, voxel size = 2 × 2 × 2 mm^3^, multi-band factor = 2.64 diffusion directions using 1000 and 0 s/mm^2^ ad b-values. The DTI acquisition time was 5 min and 31 s/5′31″. The MR protocol also included volumetric turbo field-echo T1-weighted, and fluid-attenuated inversion recovery sequences, axial turbo spin-echo T2-weighted sequence, axial susceptibility-weighted sequence.

### Neuropsychological assessment

All patients underwent a battery of neuropsychological tests [33–41], performed by a dedicated neuropsychologist (BZ), to investigate language, memory, executive functions, and attention. Each domain was explored through multiple tests (see Table 1), which proved to be sensitive in detecting cognitive impairment due to organic causes [42]. The raw scores were adjusted for age, education, and sex [43], with Italian normative data [33–41]. Mean and standard deviation of adjusted scores achieved by the patients on the neuropsychological battery tests are shown in Table 1.

**Table 1:** Mean (standard deviation) of adjusted scores achieved by frontal patients with lesions sparing or involving area 9 in neuropsychological battery tests. Between-group comparisons by t-test

Domain	Function tested	Test	F9	Fn	T^p^
Language	Verbal complex comprehension	Token Test [33]	35.44 (1.12)	35.12 (1.53)	0.66 ns
	Denomination of objects	Naming of objects [34]	45.59 (3.19)	44.62 (3.22)	0.40 ns
Memory	Verbal short-term memory	Digit span [35]	5.01 (0.89)	5.32 (0.39)	0.10 ns
	Verbal long-term memory	Auditory Verbal Learning Test [36]	40.37 (14.94)	44.58 (8.39)	0.21 ns
Attention	Selective attention and inhibitory mechanism	Stroop Test [37] (time)	20.56 (10.96)	18.69 (10.22)	0.85 ns
	Divited attention, attentional shifting	Trail Making Test [38] (b-a)	93.33 (6.66)	60.33 (38.47)	0.90 ns
Executive Verbal Functions	Verbal working memory	Digit Span Backward [39]	4.17 (0.65)	4.42 (0.84)	0.37 ns
	Lexical amplitude, lexical access, and organization	Phonemic fluency [40]	33.00 (15.98)	41.83 (14.43)	0.30 ns
	Semantic access and organization	Semantic fluency [40]	46.37 (18.71)	44.38 (8.40)	0.98 ns
Executive Non-Verbal Functions	Abstract non-verbal thinking and categorization	Weigl Test [41]	12.87 (3.38)	14.30 (1.40)	1.40 ns

*F9* patients with lesions involving area 9, *Fn* patients with lesions sparing area 9, *ns* not significant

**Fig. 1:**
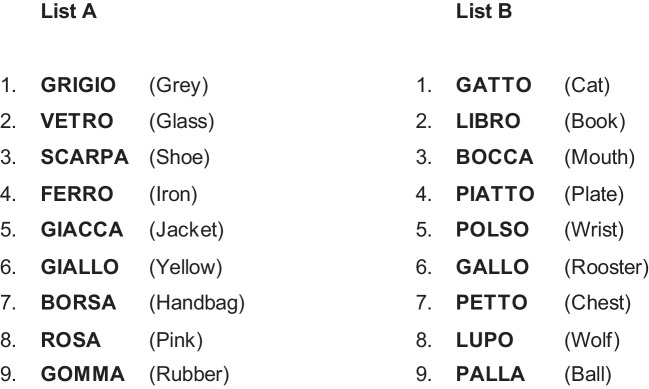
Experimental word lists used for the neuropsychological testing

The learning process was studied using two lists of words, designed by Faglioni and colleagues [44] specifically to evaluate category clustering ability (see Fig. 1).

Both lists consisted of nine disyllabic common nouns chosen from three semantic categories (animals, objects, and human body for List A; colors, materials, and clothing for list B); the nouns occur in a pseudo-random order such that no two words from the same category are next to each other. Words were balanced for frequency of use according to Bortolini and colleagues [45], across categories within each list. Imageability and familiarity ratings were equivalent in each list.

Subjects had to learn each list through fifteen study-recall trials, following Buschke and Fuld’s selective reminding technique [46]. The large number of trials enhances the likelihood of uncovering the categories within the list, a phenomenon anticipated to happen especially after several attempts both in the normal subject and, even more so, in the frontal patient. The constrained number of words in each list is meant to limit the duration of the test, as a higher cognitive demand (meaning a higher number of words) over a long period of time might overload sustained attention, thus interfering with the result.

The selective reminding technique, already used to investigate different clinical conditions using both categorically related and unrelated words, was preferred over the standard serial presentation of all the nine items before each recall trial because it allows to compensate for primacy and recency effects. Subjects were informed that the entire list would be presented on the first study trial, but only the words they had failed to recall would be reminded in each subsequent study trial; if they recalled the entire list, they would have to repeat it again on the next trial, and the examination would not stop until the 15th attempt. After that, the examiner read aloud the words at a rate of one word every two seconds, then he/she asked the subject to repeat all the words they could remember, regardless of the order of presentation. Unlimited time was allowed for recall so as not to penalize subjects with a long recall latency. The examiner recorded the order of the responses given at each recall trial.

All subjects were tested under two different verbal learning conditions (un-cued condition and cued condition) using two different lists.

In the un-cued condition, participants were not warned that a semantic structure was embedded in the list. In the cued condition, the examiner provided orally the names of the three categories by which subjects could group the nine items and then he/she wrote the category names on a piece of paper and gave it to the subjects, making sure they could see it for the whole duration of the test. Subjects were subsequently required to use categories during the recall. Lastly, the examiner began reading the items of the list.

The un-cued condition always preceded cued condition to avoid the effect of the cue on both tests. To avoid internal list effects, half of the subjects from each group studied List A in the un-cued condition, whereas the other half studied List B. In the following cued condition, each subject studied the remaining list.

Each learning test lasted no more than 30 min. All subjects performed other neuropsychological tests for 1 h between the two testing sessions.

Two main parameters were used to evaluate subjects’ performances in each list: Learning Score (LS) and Clustering Index (CI). LS constitutes the total number of recalled list words, whereas CI is calculated as the ratio between categorized words and total recalled words. A word is labeled as categorized if preceded or followed by other words pertaining to the same semantic category.

**Fig. 2:**
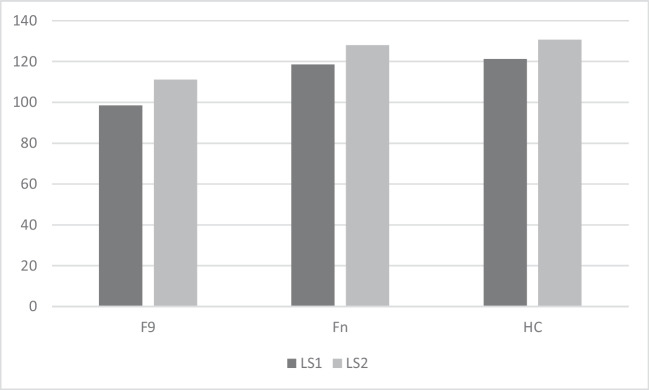
Learning performance of frontal patients with lesions sparing or involving area 9 and healthy control subjects. LS1 = Learning score in the un-cued condition, LS2 = Learning score in the cued condition, F9 = patients with lesions involving area 9, Fn = patients with lesions sparing area 9, HC = healthy control subjects

**Fig. 3:**
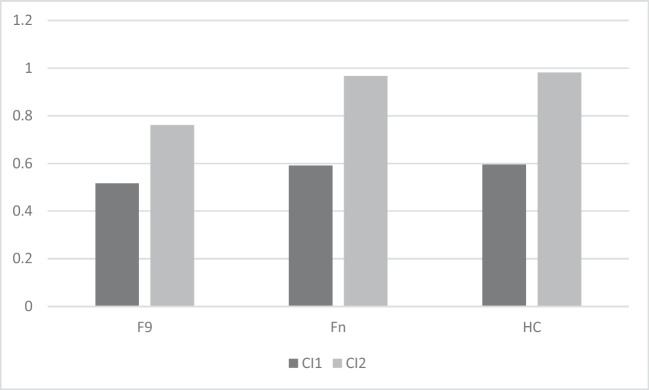
Semantic categorization ability of frontal patients with lesions sparing or involving area 9 and healthy control subjects. CI1 = Clustering Index in the un-cued condition, CI2 = Clustering Index in the cued condition, F9 = patients with lesions involving area 9, Fn = patients with lesions sparing area 9, HC = healthy control subjects

**Fig. 4:**
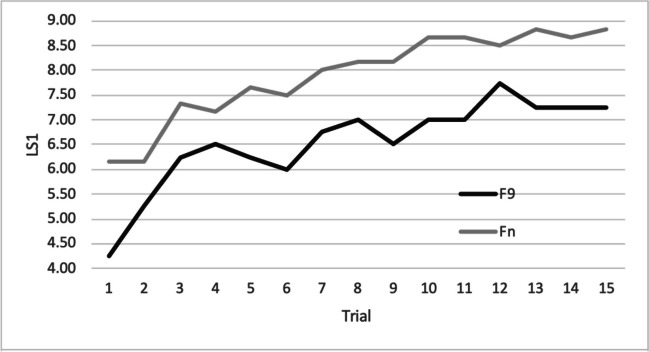
Learning curve of frontal patients for the uncued condition. LS1 = Average number of words recalled, F9 = patients with lesion involving area 9, Fn = patients with lesion sparing area 9

**Fig. 5:**
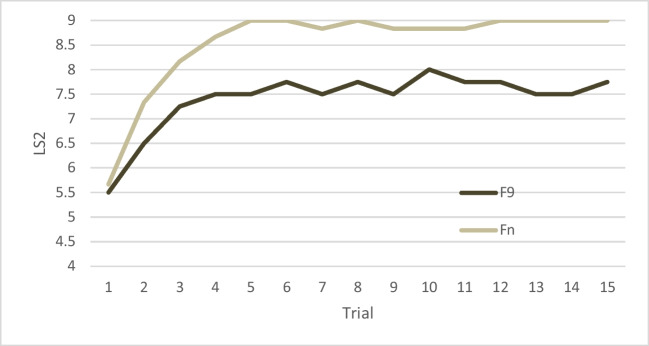
Learning curve of frontal patients for the cued condition. LS2 = Average number of words recalled, F9 = patients with lesion involving area 9, Fn = patients with lesion sparing area 9

Mean and standard deviation of LS and CI for Fn, F9 and HC are shown in Table 2 and represented in Figs. 2 and 3. Average number of words recalled by frontal patients at each trial in the uncued and cued condition are shown in Figs. 4 and 5.

**Table 2:** Mean (standard deviations) of Learning Scores and Clustering Index achieved by frontal patients with lesions sparing or involving area 9 and healthy control subjects in the un-cued and cued condition

	F9	Fn	HC
LS1	98.5 (36.72)	118.5 (6.92)	121.2 (11.15)
LS2	111 (39.44)	128 (3.63)	130.6 (3.56)
CI1	0.517 (0.136)	0.591 (0.123)	0.594 (0.217)
CI2	0.761 (0.406)	0.968 (0.024)	0.982 (0.023)

*LS1* learning score in the un-cued condition, *LS2* learning score in the cued condition, *CI1* clustering Index in the un-cued condition, *CI2* clustering Index in the cued condition, *F9* patients with lesions involving area 9, *Fn* patients with lesions sparing area 9, *HC* healthy control subjects

### Statistical analyses

Mixed models were adopted to test the effect of *Group*, *Condition* and their interaction on the CI and LS, by addressing *Subject* as the cluster.

**Fig. 6:**
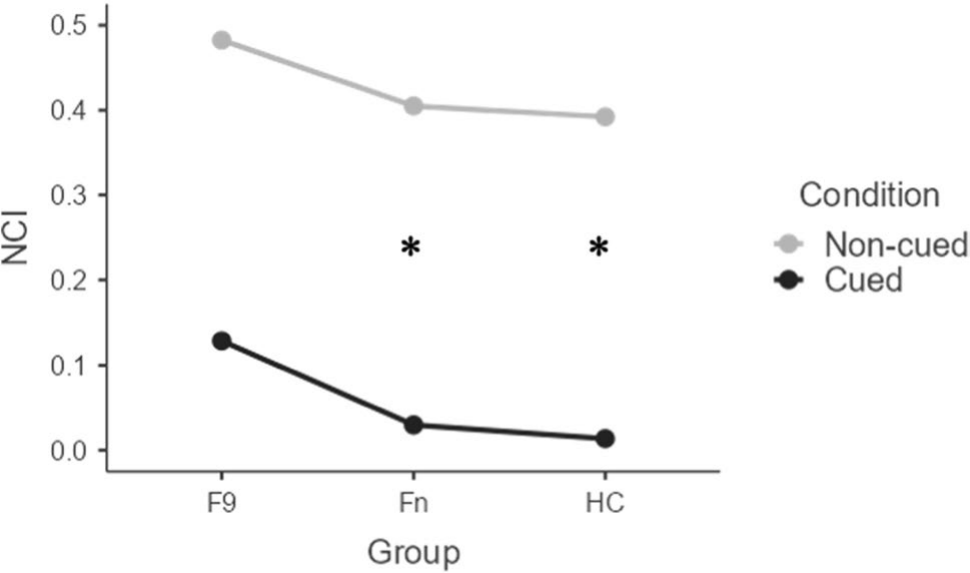
Graphical decomposition of the Group*Condition interaction on the NCI. NCI = Non-Categorization Index, F9 = patients with lesions involving area 9, Fn = patients with lesions sparing area 9, HC = healthy control subjects, * = significant between-condition difference

Both outcomes proved not to be Normally distributed, as indexed by significant Shapiro–Wilk’s statistics (*p*s < 0.001) and graphical inspections (*i.e.*, histograms and quantile–quantile plots) on raw variables – the latter revealing heavily left-skewed and overdispersed distributions. Both the CI and the LS were thus reversed, by subtracting values from their theoretical maximum (*i.e.*, 1 for the CI and 135 for the LS) – in order for them to be addressed as outcomes within generalized linear mixed models (GLMMs) assuming heavily right-skewed, overdispersed distributions [47]. More specifically, the reversed CI and LS – *i.e.*, the Non-Categorization Index (NCI) and the number of unrecalled words (UWs) – were assumed to underlie a Gamma [48] and a Negative Binomial [49] distribution, respectively – as the former models continuous, and the latter frequency, data. Since the three groups were matched for age (*F*(2,17) = 0.01; *p* = 0.990), education (*F*(2,17) = 0.66; *p* = 0.529) and sex (χ^2^(2) = 1.67; *p* = 0.435), none of these variables was entered as a covariate into the abovementioned models. Results of the analysis were shown in Fig. 6.

The significance level was set at α = 0.05; within both the Gamma and the Negative Binomial GLMM, *post-hoc* comparisons were Bonferroni-corrected. Analyses were run via jamovi 2.3 (https://www.jamovi.org).

## Results

The Gamma GLMM on the NCI revealed a significant main effect of *Condition* (χ^2^(1) = 40.62; *p* < 0.001), with the non-cued condition resulting in higher NCI values (*M* = 0.43; *SE* = 0.10) when compared to the cued one (*M* = 0.04; *SE* = 0.01), and *Group* (χ^2^(2) = 6.66; *p* = 0.036), whose decomposition revealed that F9 patients (*M* = 0.25; *SE* = 0.11) performed worse (*p* = 0.030) than HC (*M* = 0.07; *SE* = 0.02), with no other comparison yielding significance (*p*s ≥ 0.365). Moreover, the significant Group*Condition interaction (χ^2^(2) = 7.63; *p* = 0.022) (see Fig. 6) revealed that the cued condition selectively favored HC (cued: *M* = 0.01; *SE* = 0.01 *vs.* non-cued: *M* = 0.39; *SE* = 0.12; *p* < 0.001) and Fn patients (cued: *M* = 0.03; *SE* = 0.01 *vs.* non-cued: *M* = 0.41; *SE* = 0.16; *p* < 0.001), but not F9 patients (cued: *M* = 0.13; *SE* = 0.08 *vs.* non-cued: *M* = 0.48; *SE* = 0.23; *p* = 1).

As for the Negative Binomial GLMM on UWs, a main effect of *Condition* (χ^2^(2) = 19.28; *p* < 0.001) – with the non-cued condition overall resulting in a higher number of UWs (*M* = 17.3; *SE* = 6.07) when compared to the cued one (*M* = 6.91; *SE* = 1.39) – and *Group* (χ^2^(1) = 6.32; *p* = 0.042) emerged – with the only significant *post-hoc* comparison (*p* = 0.038) being that between F9 (*M* = 18.37; *SE* = 6.07) and HC (*M* = 6.91; *SE* = 1.5). At variance with the results on the NCI, the Group*Condition interaction on UWs was not significant (χ^2^(2) = 0.53; *p* = 0.767).

Performance on the neuropsychological battery tests did not differ between F9 and Fn, ruling out the influence of other cognitive disorders on the above comparisons. The ability of frontal patients to perform highly on the Auditory Verbal Learning Test indicates that their deficit is confined to the semantic organization of verbal material and does not involve other aspects of memory functioning.

## Discussion

In the non-cued word list learning task, F9 recall fewer words and they are also less likely to group them into categories when compared to HC. However, Fn do not significantly differ from HC in terms of number of recalled words or propensity to spontaneously categorize items.

One may theorize that the identification of categories is quite demanding and could overload WM, slowing down HC’ efficiency in the subsequent trials. Instead, frontal patients’ WM – even if generally impaired because of the lesion – is not overloaded by the identification of categories since patients tend to neglect them. This could explain why the differences in performance between patients and controls are reduced, remaining evident only for F9. However, in line with Alexander and colleagues’ [5] observation, A9 would play a critical role in learning process.

Even when given the strategic cue, F9 patients exhibit a decrease in word acquisition and categorization, while Fn patients show no difference from HC. Previous studies showed that strategic cues improve learning in frontal patients, who may reach normal scores [6, 8, 10]. However, these studies did not design subgroups according to the frontal lesion site, which is a relevant factor for subjects’ performance. In our study, this subdivision showed that, in the case of A9 involvement, the strategic cue does not improve learning.

It is remarkable that, compared to HC, F9 do not benefit from the semantic cue in organizing recalled words, unlike Fn who, similarly to HC, significantly improve when transitioning from the no-cued to the cued condition. This confirms the crucial role played by area 9 in the implementation of learning strategies, as suggested by TMS evidence [27].

The small sample size and the lack of specific neuroimaging data impose some caution in the interpretation of the results; however, the significance of the statistical comparisons in line with the experimental hypothesis encourages further studies on this topic.

In summary, our results show that frontal patients’ verbal learning defect is due to impaired categorical strategy implementation and that this ability can be specifically attributed to the left area 9.

## Data Availability

Datasets cannot be made publicly available on both ethical and legal grounds but may be made available upon reasonable request of interested researchers to the Corresponding Author, who will in turn forward a request for a data transfer agreement to the relevant Ethical Committee.

## Abbreviations

FL: Frontal lobe-damaged patients
Fn: Patients with lesion sparing area 9
F9: Patients with lesion involving area 9
HC: Healthy control
PFC: Prefrontal cortex
VLPFC: Ventrolateral prefrontal cortex
DLPFC: Dorsolateral prefrontal cortex
STM: Short-term memory
WM: Working memory
BA9: Area 9 according to Brodmann’s classification
A9: Area 9 according to Petrides and Pandya’s classification
LS: Learning Score
CI: Clustering Index
NCI: Non-Categorization Index
UWs: Unrecalled words
