# Correction to: Verbal learning in frontal patients: area 9 is critical for employing semantic strategies

**DOI:** 10.1007/s10072-024-07596-4

**Published:** 2024-05-20

**Authors:** Alessandro Cocuzza, Giulio Bertani, Giorgio Conte, Edoardo Nicolò Aiello, Barbara Zarino, Teresa Difonzo, Stefano Zago, Leonardo Tariciotti, Claudia Gendarini, Elena Baratelli, Federico Verde, Barbara Poletti, Nicola Ticozzi, Mauro Pluderi, Marco Locatelli, Giacomo Pietro Comi, Maria Cristina Saetti

**Affiliations:** 1 Department of Pathophysiology and Transplantation, University of Milan, 20122 Milan, Italy; 2 Foundation IRCCS Ca’ Granda Ospedale Maggiore, Policlinico, Neurology Unit, 20122 Milan, Italy; 3 Foundation IRCCS Ca’ Granda Ospedale Maggiore, Policlinico, Neurosurgery Unit, 20122 Milan, Italy; 4 Foundation IRCCS Ca’ Granda Ospedale Maggiore, Policlinico, Neuroradiology Unit, 20122 Milan, Italy; 5 Department of Neurology and Laboratory of Neuroscience, IRCCS Istituto Auxologico Italiano, Milan, Italy; 6 Humanitas Psico Medical Care, MCH SRL, Via Manzoni, 113, Rozzano, 20089 Milan, Italy; 7 Department of Neurology, G. Salvini Hospital, Garbagnate Milanese, Italy; 8 Department of Oncology and Hemato‑Oncology, Universitàdegli Studi di Milano, Milano, Italy

**Correction to** **: Neurological Sciences (2024)**

10.1007/s10072-024-07569-7

The Abstract of the original article needs an update. The updated abstract as follows:

**Abstract**

**Introduction** Learning is a long-term memory process heavily influenced by the control processes implemented by working memory, including recognition of semantic properties of items by which subjects generate a semantic structure of engrams.

**Aim** The aim of this study is to investigate the verbal learning strategies of patients affected by a tumor in the left frontal lobe to highlight the role of area 9.

**Method** Ten patients with frontal low-grade gliomas and ten healthy control subjects, matched for age, sex and education, were recruited and then evaluated with a two-part verbal learning test: multi-trial word list learning in free recall, and multi-trial word list learning preceded by an explicit semantic strategy cue. Frontal patients were divided into two groups: those either with frontal lesions involving or sparing area 9.

**Results** In comparison to healthy control subjects, frontal patients with lesions involving area 9 memorized fewer words and displayed difficulty in using semantic strategies. When the strategy was suggested by the examiner, their performance improved, but to a lesser extent than the healthy control. Conversely, frontal patients with lesions sparing area 9 showed similar results to healthy control subjects.

**Conclusion** The results suggested that, while the identification of the categorical criterion requires the integrity of the entire dorsolateral prefrontal area, only area 9, and not the surrounding areas, could be responsible for the effective use of semantic strategies in learning tasks.

The original article has been corrected.

